# Monolayer MoS_2_ on Paper with a Copper Electrode: An Integrated CVD-Grown Broadband UV–Visible Photodetector

**DOI:** 10.3390/mi17101156

**Published:** 2026-09-30

**Authors:** Amal Alzahrani, Maha Alaqeel, Mohamed Nejib Hedhili, Yaping Zhang, Roaa Sait, Reem Altuwirqi, Hala Al-Jawhari, Arwa Kutbee, Areej Aljarb

**Affiliations:** 1 Department of Physics, King Abdulaziz University, Jeddah 21589, Saudi Arabia; aalzahrani3995@stu.kau.edu.sa (A.A.); alaqeelmaha.a@gmail.com (M.A.); ramsait@kau.edu.sa (R.S.); raltuwirqi@kau.edu.sa (R.A.); hala.aljawhari@gmail.com (H.A.-J.); 2 Core Labs, King Abdullah University of Science and Technology (KAUST), Thuwal 23955-6900, Saudi Arabia; mohamed.hedhili@kaust.edu.sa (M.N.H.); yaping.zhang@kaust.edu.sa (Y.Z.); 3 Center of Nanotechnology, King Abdulaziz University, Jeddah 21589, Saudi Arabia

**Keywords:** monolayer MoS_2_, broadband photodetectors, Cu electrode, two-dimensional material, flexible photodetectors

## Abstract

The commercial viability of flexible and disposable photodetectors is often constrained by the reliance on costly noble-metal electrodes and conventional substrates. Here, we address these challenges by integrating a chemical vapor deposition (CVD)-grown monolayer MoS_2_ with a low-cost copper (Cu) electrode on a paper substrate, combining scalable semiconductor synthesis with inexpensive device components. Raman, photoluminescence, and X-ray photoelectron spectroscopy measurements confirmed the crystallinity of the MoS_2_ monolayer and its direct bandgap of 1.8 eV. The fabricated device demonstrated a broadband photoresponse covering the ultraviolet and visible regions, achieving a maximum responsivity and specific detectivity of 1.37 mA/W and 3.17 × 10^9^ Jones, respectively, at 320 nm under 5 V bias and an incident power density of 2.14 mW/cm^2^. The device retains a well-resolved photoresponse under bending to a radius of 11.25 mm and over 100 bending cycles, confirming the mechanical robustness of the paper platform. By replacing the noble-metal contacts with low-cost Cu electrodes and conventional polymer substrates with paper while employing scalable CVD-grown MoS_2_, this work establishes an economically viable platform for the large-scale manufacture of flexible and disposable photodetectors while providing a scalable pathway toward their large-area manufacture.

## 1. Introduction

In modern optoelectronic systems, broadband photodetectors play a crucial role in applications such as imaging [1], UV sensing [2], security applications [3], and optical communication [4]. Conventional photodetectors are predominantly based on bulk materials such as Si [5], InGaAs [6], and ZnO [7]; however, these materials involve complex fabrication processes and offer limited mechanical flexibility, and some also entail high material and production costs [8,9,10]. These limitations hinder their adoption in emerging applications that demand lightweight, flexible, and disposable devices. Consequently, there is a growing interest in developing photodetectors that combine high optoelectronic performance with low manufacturing cost [11,12]. Achieving this objective requires optimization of the complete device architecture, including the photoactive semiconductor, the substrate, and the electrode.

Among the three principal components of a photodetector, the photoactive semiconductor largely determines the optical response of the devices. Consequently, two-dimensional (2D) materials have attracted considerable attention because they combine outstanding optoelectronic properties with the potential for scalable synthesis. Unlike zero-band-gap graphene which is the first 2D material that used in photodetector applications [13], transition metal dichalcogenides (TMDs), particularly molybdenum disulfide (MoS_2_), have emerged as promising photoactive materials owing to their thickness-dependent band gap, strong light–matter interaction, and excellent optical absorption [14,15]. Since the first MoS_2_ photodetector was demonstrated in 2012 [16], continuous advances in material synthesis, device engineering, and scalable manufacturing have established MoS_2_ as one of the most extensively investigated semiconductors for next-generation photodetectors [8,17]. This interest reflects the layered structure of MoS_2_, in which, as in other TMDs, strong covalent bonds within each layer coexist with weak van der Waals interactions between the layers [14]. In particular, chemical vapor deposition (CVD) enables the reproducible growth of high-quality monolayer MoS_2_ over wafer-scale areas, providing a scalable manufacturing route for next-generation electronic and optoelectronic devices [18,19].

While advances in CVD-grown MoS_2_ have addressed the scalability of the photoactive semiconductor, the substrate and electrode remain two major contributors to the overall cost and manufacturability of flexible photodetectors. Most reported MoS_2_ photodetectors continue to rely on rigid substrates such as Si and sapphire [20,21], which can limit device scalability and environmental sustainability. Moreover, the increase in electronic waste, projected to reach 74 million tons by 2030 [22], necessitates the development of an eco-friendly electronic platform. To address these challenges, flexible and sustainable substrates, such as paper, have recently gained attention due to their low cost and lightweight properties [23,24]. In parallel, many reported MoS_2_ photodetectors employ noble-metal electrodes, particularly Au and Ag [25,26], owing to their high conductivity and favorable contact properties. In addition, the lower adhesion energy in the Au–MoS_2_ interface, prompting the use of additional material such as Ti and Cr that may be time-consuming and complicates the process [27,28]. Therefore, replacing expensive noble metals with cost-effective alternatives such as copper (Cu) offers a further pathway to reduce the fabrication cost while retaining high conductivity.

Each of these three elements has already been demonstrated individually: CVD-grown monolayer MoS_2_ as a high-quality ultraviolet–visible (UV–Vis) photoactive layer [20,29,30], paper as a low-cost and flexible substrate for MoS_2_ photodetectors [31,32], and copper as an inexpensive, non-noble contact for flexible MoS_2_ devices [31,33]. However, to the best of our knowledge, these complementary approaches have not yet been integrated into a unified device architecture; specifically, no prior report combines CVD-grown monolayer MoS_2_, a paper substrate, and a Cu electrode in a single device while preserving the quality of the monolayer through transfer. Addressing this gap is an important step toward the realization of a genuinely low-cost, scalable, and sustainable 2D optoelectronic platform.

In this work, we address this challenge by demonstrating a broadband photodetector that integrates monolayer MoS_2_ on a paper substrate with a cost-effective copper (Cu) electrode as an alternative to noble-metal contacts. Monolayer MoS_2_ was synthesized via CVD, transferred onto paper using polydimethylsiloxane (PDMS), and contacted with a Cu electrode deposited by magnetron sputtering, providing a low-cost and paper-compatible process that also enables a sustainable alternative to noble-metal contacts. The crystallinity, monolayer thickness, and chemical state of the films were confirmed by Raman, photoluminescence, and X-ray photoelectron spectroscopy, and the optical bandgap was extracted from UV–Vis absorption. We further observe that the paper-based device exhibited a higher photocurrent and responsivity than the as-grown device on sapphire. Building on this improved platform, optoelectronic measurements across six wavelengths along the UV and visible regions show broadband photodetection, with the strongest response in the UV at 320 nm, reaching a responsivity of 1.37 mA/W and a specific detectivity of 3.17 × 10^9^ Jones.

## 2. Experimental Details

Monolayer MoS_2_ Synthesis. Monolayer MoS_2_ was grown on sapphire via CVD using powder precursors. Molybdenum trioxide (MoO_3_, Sigma-Aldrich, St. Louis, MO, USA, 99.5% purity) and sulfur (S, Sigma-Aldrich, 99.5% purity) were employed as source precursors. Following the method described in [34,35], 4 g of sulfur powder was placed in the upstream zone of the furnace, while 0.8 g of MoO_3_ powder was placed downstream adjacent to the 2-inch sapphire substrate. The growth process was initiated by gradually heating the center of the furnace to 750 °C and maintaining this temperature for 17 min, then increasing to 800 °C for 7 min. On the other hand, the upstream was maintained at 160 °C with Argon (Ar) flow rate of 70 sccm throughout the process. Finally, the furnace was naturally cooled down to room temperature.

MoS_2_ Transfer and Device Fabrication. A glossy paper was used as a substrate for the device. By using PDMS, a layer was applied to the MoS_2_/sapphire. Subsequently, with the assistance of DI water drops, the PDMS/MoS_2_ layer was peeled off the sapphire and placed onto the paper. To improve adhesion and transfer quality, the PDMS/MoS_2_ stack was left on paper under a vacuum overnight, then the PDMS was peeled off, leaving the MoS_2_ on the paper. The same procedure was also used to transfer the monolayer onto a fresh, bare sapphire substrate, providing a substrate-matched control in which the as-grown and transferred films differ only by the transfer process (Supplementary Figure S1). The device was first measured with the full electrode array and then cut to a single electrode pair; the I-V and I-T characteristics were unchanged after cutting (Supplementary Figure S2), confirming that adjacent structures do not contribute to the measured signal. Finally, the Cu electrode was deposited onto the MoS_2_/paper by sputtering (Angstrom Engineering AMOD system) through a square shadow mask of 1.2 mm line width and 1 mm line spacing, at an applied power of 40 W and a working pressure of 3 mTorr for 15 min, giving a Cu thickness of 44 nm.

Structural and Optical Characterization. To investigate the optical properties, Raman and photoluminescence (PL) were performed using a Witec Alpha 300 system with a 532 nm laser excitation source. To further examine the chemical composition and bonding states, X-ray photoelectron spectroscopy (XPS) measurements were performed using a Karos Axis Supra DelayLine Detector (DLD) spectrometer (Kratos Analytical Ltd., Manchester, UK) equipped with a monochromatic Al Kα X-ray source (*hυ* = 1486.6 eV) at 150 W. In addition, the UV–Vis absorption spectrum was recorded using a Thermo Scientific spectrometer (Thermo Fisher Scientific, Waltham, MA, USA) over a wavelength range from 190 nm to 1100 nm.

Optoelectronic Characterization. Electrical measurements of the devices were performed using a SemiProbe probe station connected to a Keithley 4200-SCS instrument (Keithley Instruments, Cleveland, OH, USA) at room temperature. All measurements were carried out under dark and illuminated conditions at six wavelengths: 320, 420, 490, 530, 580, and 590 nm. Illumination was provided by a 300 W xenon lamp (MAX-303, Asahi Spectra, Tokyo, Japan) with bandpass filters (FWHM 10 ± 2 nm) and, at 590 nm, a long-pass filter transmitting 590–600 nm. The collimated beam illuminated a 23 mm × 23 mm area, much larger than the device active area. Additionally, the incident power density was calibrated using a Newport 1918-R power meter (Newport Corporation, Irvine, CA, USA).

Mechanical flexibility was assessed by conforming the device to curved formers giving bending radii of 12.5, 11.25 and 10 mm, and by repeated flat–bent–flat cycling at a fixed radius of 12.5 mm for up to 100 cycles, with the illuminated area bent in every case. The photoresponse under bending and after cycling was recorded under 320 nm illumination at an incident power density of 2.14 mW/cm^2^ and an applied bias of 5 V, the same bias as used for the unbent device characterization. Humidity exposure was carried out using an ultrasonic diffuser filled with deionized water. The device was first dried with a nitrogen gun and its photoresponse was recorded as the dry reference. It was then held in the diffuser mist with tweezers, so that both faces of the sample were exposed, for humid exposure times of 30 s, 60 s and 180 s; the sample was not dried between steps. After each step the sample was removed from the mist and its current-time response under 320 nm illumination was recorded at 10 V and 5.5 mW/cm^2^, the same conditions as the dry reference measurement. All measurements were performed in air at room temperature.

The performance of the devices was evaluated using standard figures of merit, including photocurrent, responsivity, specific detectivity, and noise equivalent power:

Photocurrent, *I_ph_* = *I_l_* − *I_d_* (1)

(2) $$\mathrm{Responsivity},\;R=\frac{I_{\mathrm{ph}}}{P_{\mathrm{opt}}}$$

(3) $$\mathrm{Specific}\;\mathrm{Detectivity},\;D^{*}=R\sqrt{\frac{A}{2eI_{d}}}$$

(4) $$\mathrm{Noise}\;\mathrm{Equivalent}\;\mathrm{Power}\;(\mathrm{NEP})=\frac{i_{n}}{R}$$

where *A* is the active area, *I_l_* is the illuminated current, *I_d_* is the dark current, *P_opt_* is the incident optical power (*P_opt_* = incident power density × active area), *e* is the electron charge, and *i_n_* is the noise current. The noise current was taken as the shot-noise limit of the dark current, *i_n_*$=\;\sqrt{2qI_{d}}$, and the active area *A* was defined by the electrode geometry as the area of MoS_2_ between adjacent Cu lines, giving 1.2 × 10^−2^ cm^2^ for both the as-grown and transferred devices.

## 3. Results and Discussion

**Figure 1:**
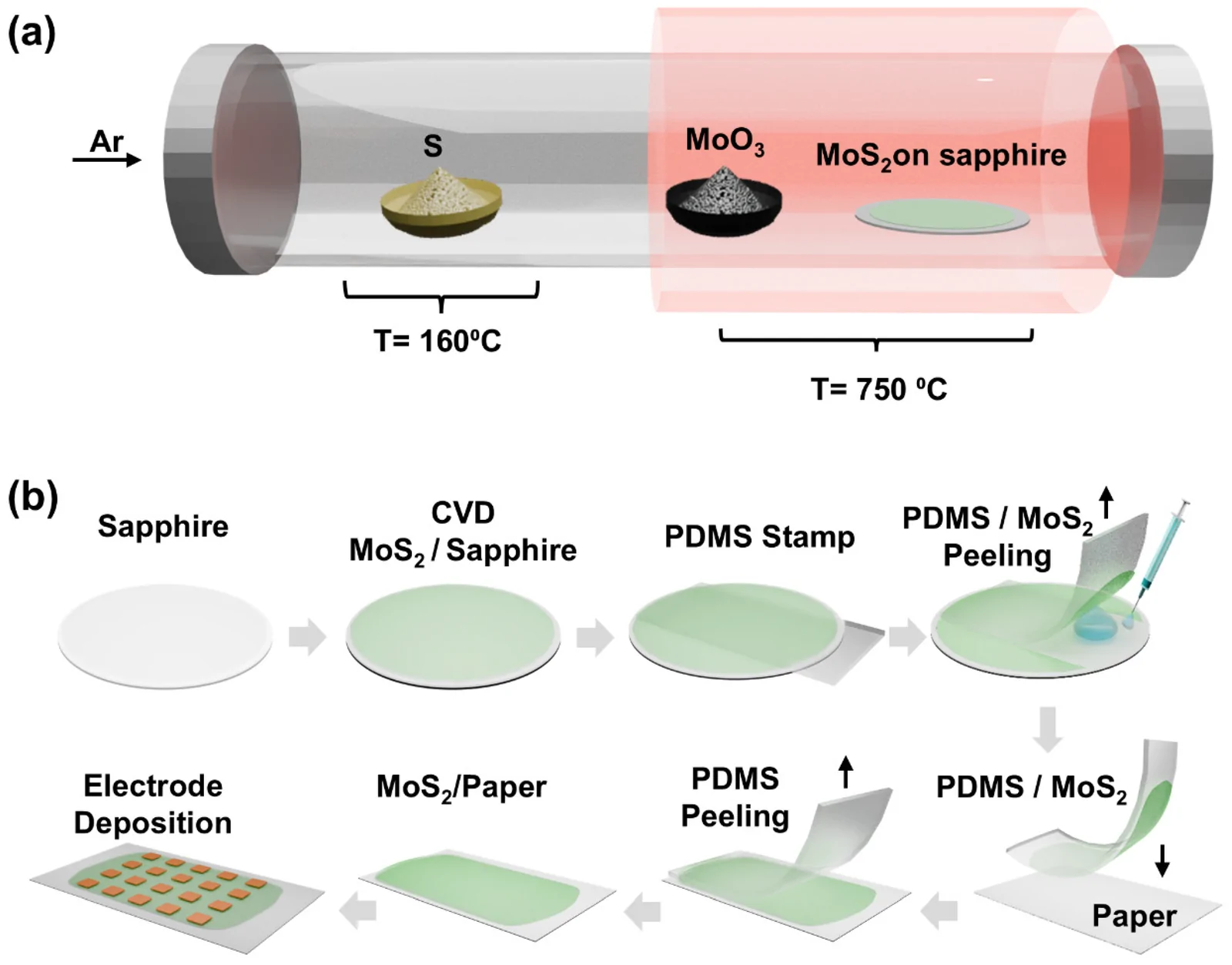
Schematic illustration of the fabrication process of the MoS_2_ paper-based photodetector. (**a**) CVD growth of monolayer MoS_2_ on a sapphire substrate using MoO_3_ and sulfur precursors. (**b**) Step-by-step PDMS-assisted transfer process of MoS_2_ from sapphire substrate to a paper substrate, followed by Cu electrode deposition. Gray arrows indicate the sequence of process steps, and black arrows indicate the direction of placing or removing the PDMS.

The overall fabrication route is summarized schematically in Figure 1 and described in detail in Section 2. As illustrated in Figure 1a, the MoS_2_ was first grown on sapphire by CVD using powder precursors. After that, the as-grown MoS_2_ monolayer was further transferred onto the paper followed by copper electrode deposition as seen in Figure 1b. By combining a CVD-grown monolayer, a low-cost paper substrate, and a non-noble copper electrode, a scalable and cost-effective route to MoS_2_-based photodetectors is obtained.

### 3.1. Structural and Optical Characterization

**Figure 2:**
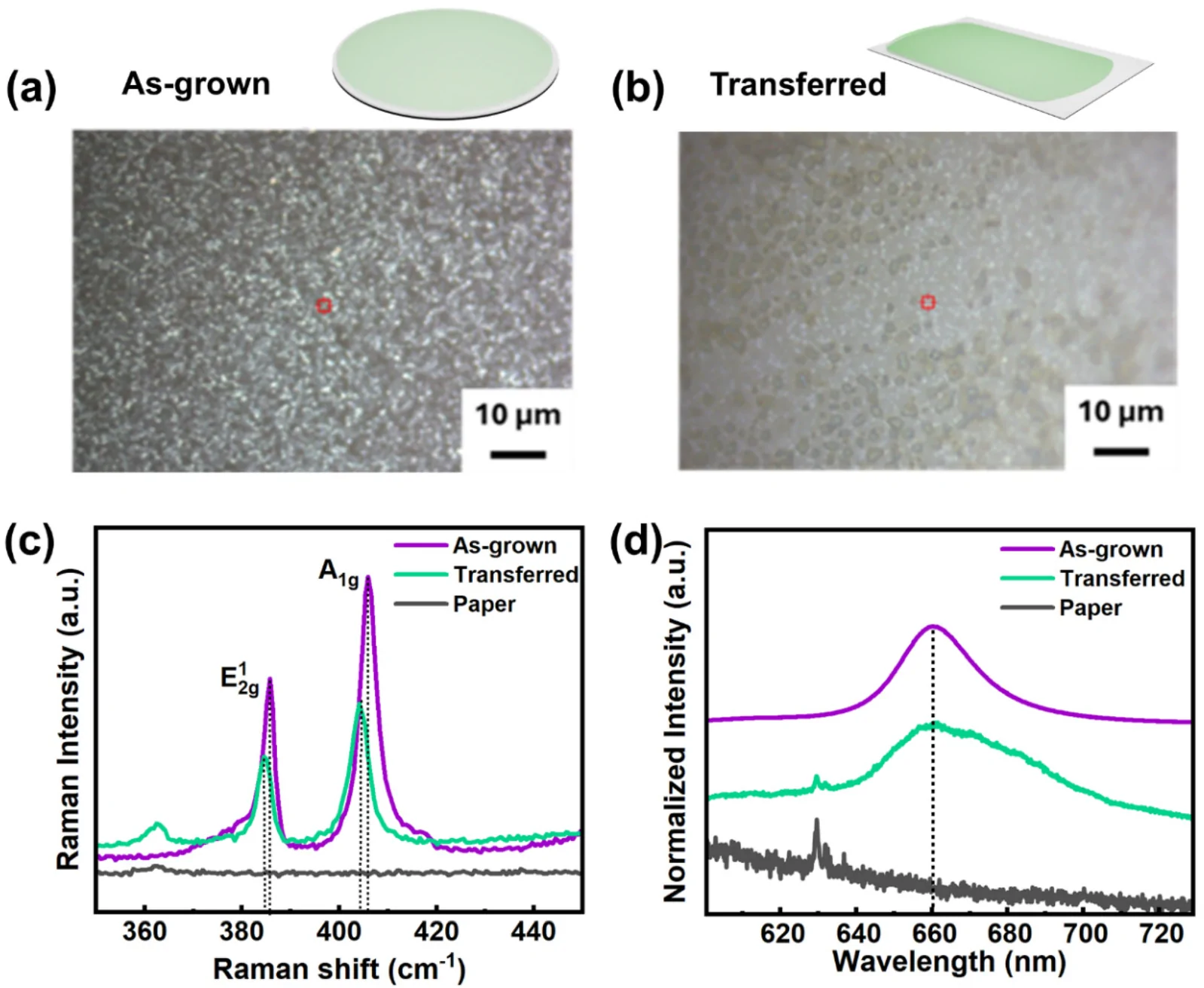
Structural and optical characterization of the CVD-grown monolayer MoS_2_ before and after transfer. (**a**,**b**) Optical images of as-grown and transferred MoS_2_ layer, respectively. (**c**) Raman spectra of the as-grown, transferred MoS_2,_ and of the bare paper. (**d**) Normalized PL spectra of the as-grown and transferred MoS_2_ and of the bare paper. Dotted lines in (**c**,**d**) indicate the peak maximum positions. The marker in the optical images indicates the position where the Raman and PL spectra were acquired.

Prior to electrical measurements, the structural and optical properties of the MoS_2_ films were characterized to ensure high-quality growth. Figure 2a,b presents the optical microscope images of the film before and after the transfer, both of which exhibit uniform coverage. Figure 2c compares the Raman spectra of the as-grown and transferred MoS_2_, which exhibit two characteristic first-order modes: the in-plane *E*^1^_2_*_g_* mode at 385.7 cm^−1^ (as-grown) and 384.5 cm^−1^ (after transfer), arising from the opposing vibration of the Mo and S atoms within the basal plane, and the out-of-plane *A*_1_*_g_* mode at 405.9 and 404.2 cm^−1^, respectively, arising from the vibration of the S atoms perpendicular to the layer. As the number of layers increases, the *A*_1_*_g_* mode stiffens while the *E*^1^_2_*_g_* mode softens, so that the frequency separation between the two modes widens systematically with the layer count and is therefore commonly used to identify the number of MoS_2_ layers [36]. Here, the separation is 20.2 cm^−1^ for the as-grown film and 19.7 cm^−1^ after the transfer onto paper, indicating monolayer thickness in both cases [20,36]. In addition, the FWHM of the *E*^1^_2_*_g_* mode, 3.4 cm^−1^, is preserved after the transfer, indicating that the high crystallinity of the MoS_2_ is retained [37]. The bare paper substrate shows no Raman modes or PL emission in the measured range, so the spectra of the transferred film originate entirely from the MoS_2_. Furthermore, from the PL spectrum in Figure 2d, we observed a sharp peak at 661.1 nm (1.88 eV), corresponding to the A-exciton, which remains at 660.4 nm after the transfer onto paper. The strong intensity and narrow width of this peak suggest a high-quality MoS_2_ monolayer [38].

**Figure 3:**
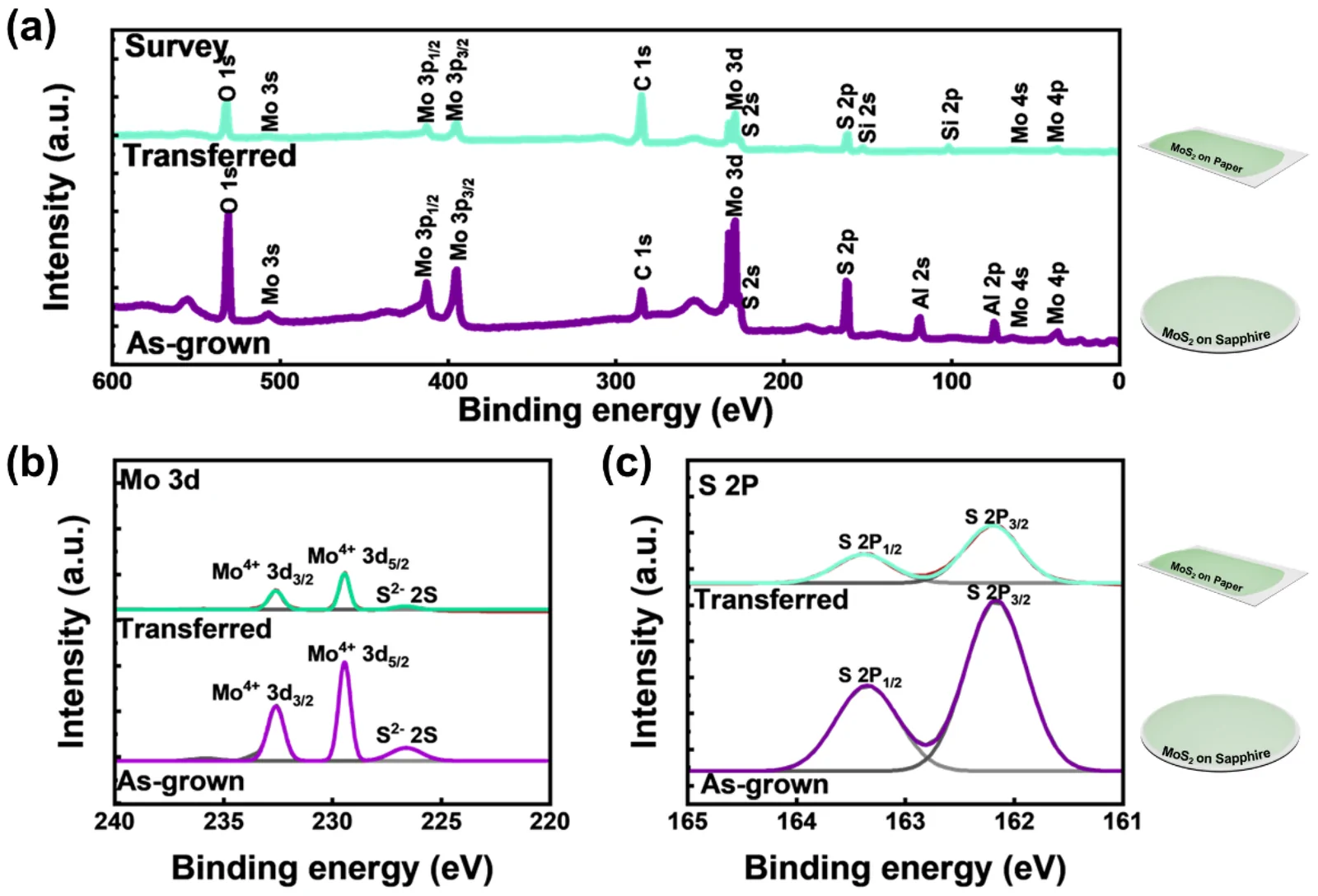
XPS analysis of monolayer MoS_2_, as-grown on sapphire and after transfer to paper. (**a**) Survey spectra; (**b**) Mo 3d core-level spectra; (**c**) S 2p core-level spectra.

While Raman and PL analyses confirm the optical properties and monolayer nature of MoS_2_, they do not provide any information about the chemical composition. Therefore, XPS was performed to verify the elemental composition and the chemical state in both the as-grown and the transferred MoS_2_ samples, as shown in Figure 3. The survey spectra in Figure 3a confirms the presence of the main constituent elements in both samples. All binding energies were calibrated against the C 1 s peak at 284.4 eV. The as-grown MoS_2_ spectrum shows signals from Mo, S, Al, and O; the Al peak refers to the sapphire substrate, and it disappears after the transfer process. Figure 3b depicts Mo 3d deconvoluted peaks of Mo 3d_3/2_ at 232.58 eV and Mo 3d_5/2_ at 229.4 eV, which are consistent with Mo^4+^ oxidation state. In the same spectrum, there is a small contribution of the S 2S peak at 226.6 eV. Figure 3c displays the typical S 2p_3/2_ at 162.17 eV and S 2p_1/2_ at 163.34 eV doublet, which refers to the sulfur oxidation state of S^2−^. These Mo 3d and S 2p peaks confirm the 2H phase of MoS_2_ [39]. As seen in Figure 3, all the peaks have the same position before and after the transfer, indicating the same stoichiometry and the preservation of the MoS_2_ film during the transfer process.

**Figure 4:**
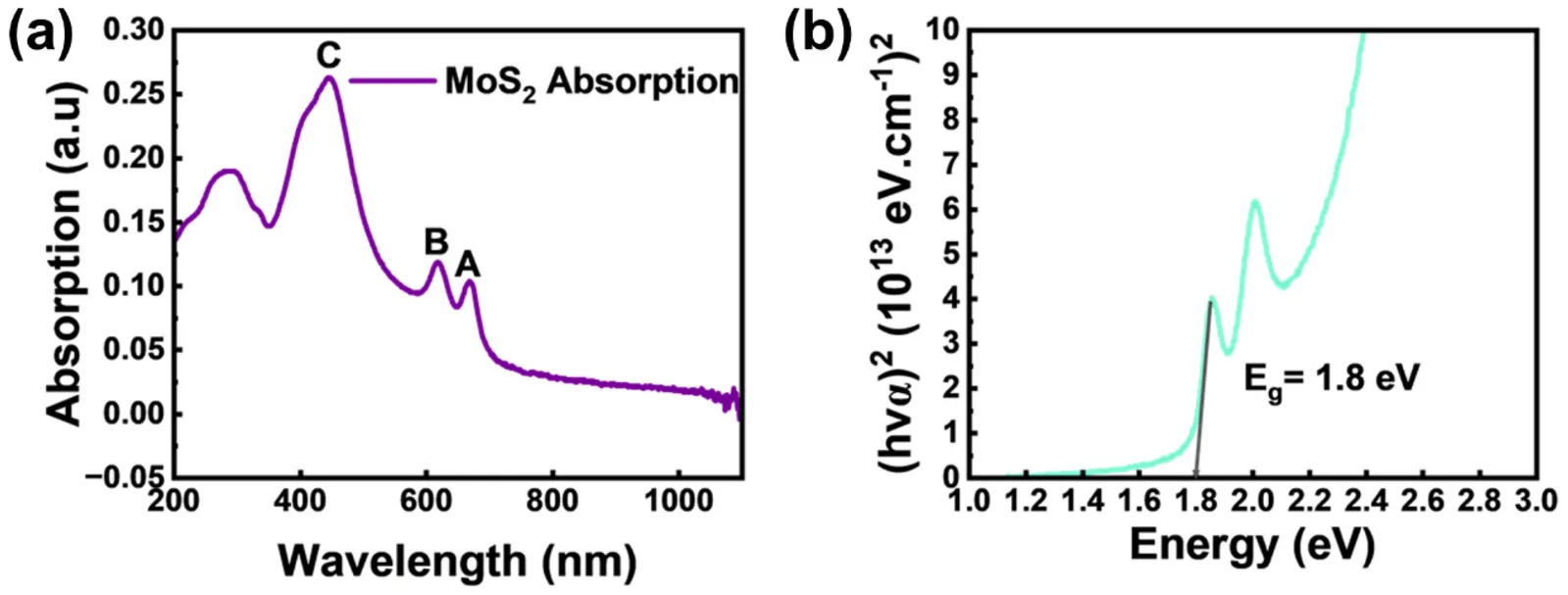
Optical absorption of the monolayer MoS_2_. (**a**) UV–Vis absorbance spectrum showing the A, B, and C excitons. (**b**) Tauc plot giving a bandgap of 1.8 eV.

To investigate the optical absorption of monolayer MoS_2_, we performed UV–Vis spectroscopy as seen in Figure 4. The absorption spectrum of the monolayer MoS_2_ film is shown in Figure 4a, obtained by subtracting the absorbance of blank sapphire. There are three peaks in the spectrum, A, B, and C, which correspond to excitons in MoS_2_. A and B excitons at 668.4 nm and 618.4 nm are the typical excitons of MoS_2_. In contrast, a broad C-exciton around 400 nm is attributed to the van Hove singularity which arises from the band nesting, where MoS_2_ has a strong excitonic response beyond its bandgap [30,40,41,42]. This spectrum clearly shows that MoS_2_ has broad absorption in the UV and visible region. In Figure 4b, the Tauc plot shows the bandgap, which is calculated to be 1.8 eV, consistent with the monolayer MoS_2_ bandgap.

### 3.2. Effect of Transfer on Photoresponse

**Figure 5:**
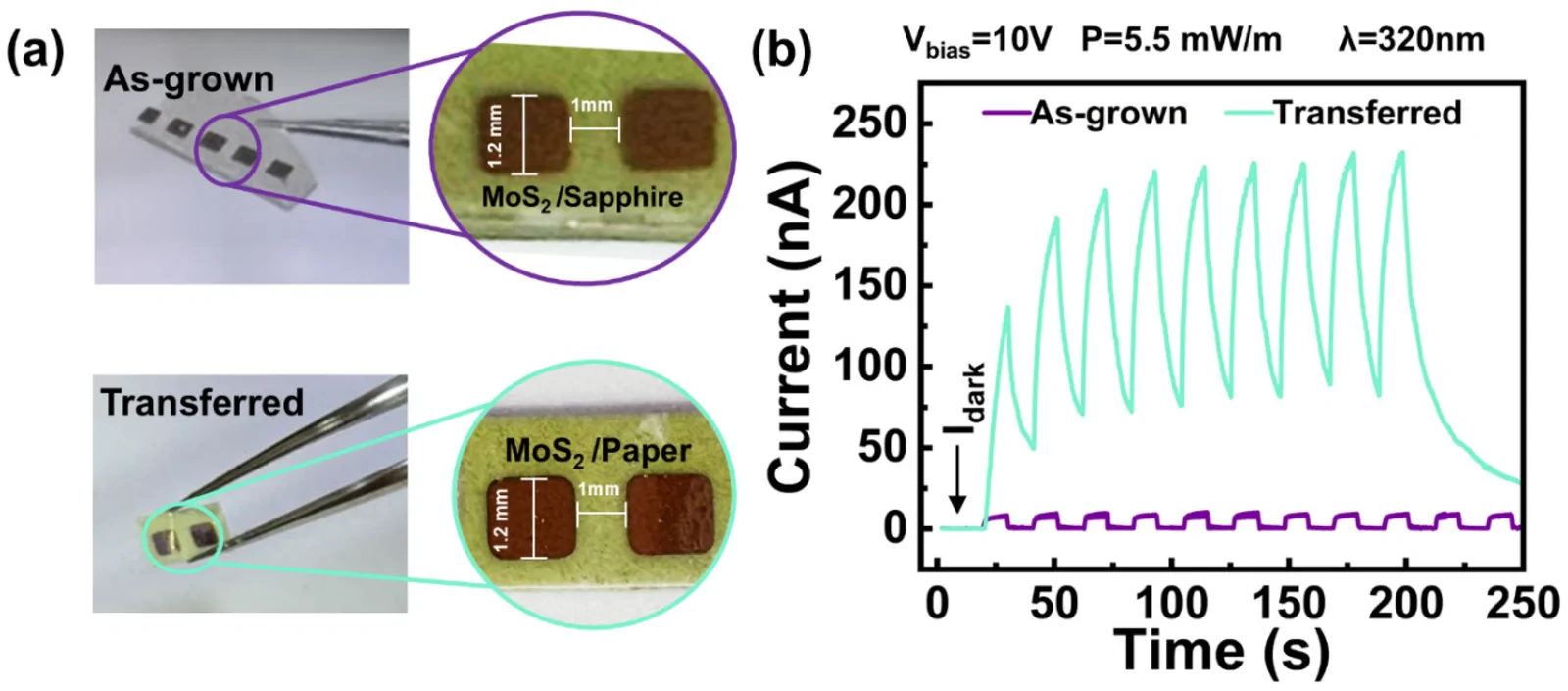
Electrical performance of the MoS_2_ device before and after transfer. (**a**) Photographs of the fabricated as-grown MoS_2_/sapphire and transferred MoS_2_/paper devices; the magnified insets show the Cu electrodes and the channel between them. (**b**) Current-time response of the as-grown and transferred devices, plotted on a common axis, under chopped 320 nm illumination at 10 V and 5.5 mW/cm^2^, including the initial dark period.

To assess how transferring the monolayer onto paper affects the photoresponse, the as-grown device (monolayer MoS_2_ on sapphire) and the paper-based device (transferred monolayer MoS_2_ on paper) were first compared, as shown in Figure 5. On the one hand, Figure 5a presents optical images of the MoS_2_ devices fabricated on sapphire and paper substrates, both using identical Cu electrodes. A control with identical Cu electrodes on bare paper shows no measurable photoresponse under 320 nm illumination (Supplementary Figure S3), confirming that the photocurrent originates from the MoS_2_ rather than from the paper substrate. On the other hand, Figure 5b compares the current-time response of the as-grown and transferred devices on a common axis under chopped 320 nm illumination at 10 V and 5.5 mW/cm^2^. Both devices show similarly low dark currents (0.484 nA on sapphire and 0.290 nA on paper), whereas the photocurrent at the first illumination peak increases from 8.435 nA for the as-grown device to 136.6 nA after transfer, an approximately 16-fold enhancement. The corresponding photo-to-dark current ratio (PDCR = *I_ph_*/*I_dark_*) increases from 17.4 for the as-grown device to 471 for the transferred device, an approximately 27-fold enhancement, owing to both the larger photocurrent and the lower dark current of the device on paper. The enhancement therefore arises under illumination rather than from an increased dark current. This comparison was recorded at 10 V and 5.5 mW/cm^2^ because the photoresponse of the as-grown device on sapphire is too weak to be resolved clearly at 5 V; both devices were measured under identical conditions. We attribute this enhancement to two sources: the release of growth-induced strain upon transfer, and trap-assisted photogating by species adsorbed at the MoS_2_/paper interface. Substrate-matched Raman measurements, in which as-grown and transferred monolayers are compared on identical sapphire substrates (Supplementary Figure S1), show a shift confined to the strain-sensitive *E*^1^_2_*_g_* mode while the doping-sensitive *A*_1_*_g_* mode remains unchanged in position, identifying strain relaxation rather than a change in carrier concentration as the responsible mechanism [36,43]; the direction of the shift is consistent with relaxation of the compressive strain imposed by the sapphire growth substrate. Because the A-exciton energy is unchanged after transfer (Figure 2d), strain relaxation acts on carrier transport rather than on light absorption: the relaxed lattice is expected to raise the carrier mobility, shortening the transit time between the electrodes and increasing the photoconductive gain [43]. The second source is evident in the transient response (Figure 5b): the transferred device shows a gradual increase in current over successive illumination cycles, indicative of persistent photoconductivity (PPC) and consistent with a trap-assisted photogating mechanism, whereby photogenerated carriers trapped at interfacial states modulate the channel conductivity and prolong the effective carrier lifetime [32]. Diffuse back-scattering from the porous paper substrate contributes secondarily: acting as an optical double-pass effect, it is bounded at approximately two-fold even for a substrate approaching unity diffuse reflectance [24,31], and therefore cannot account for the order-of-magnitude enhancement observed. Chemical modification is disfavored as a dominant cause, since the Mo 3d and S 2p core-level positions are unchanged before and after transfer (Figure 3). Based on this improvement, all subsequent measurements focused on the paper-based device to evaluate its broadband photodetection capability.

### 3.3. Broadband Photodetection Performance

**Figure 6:**
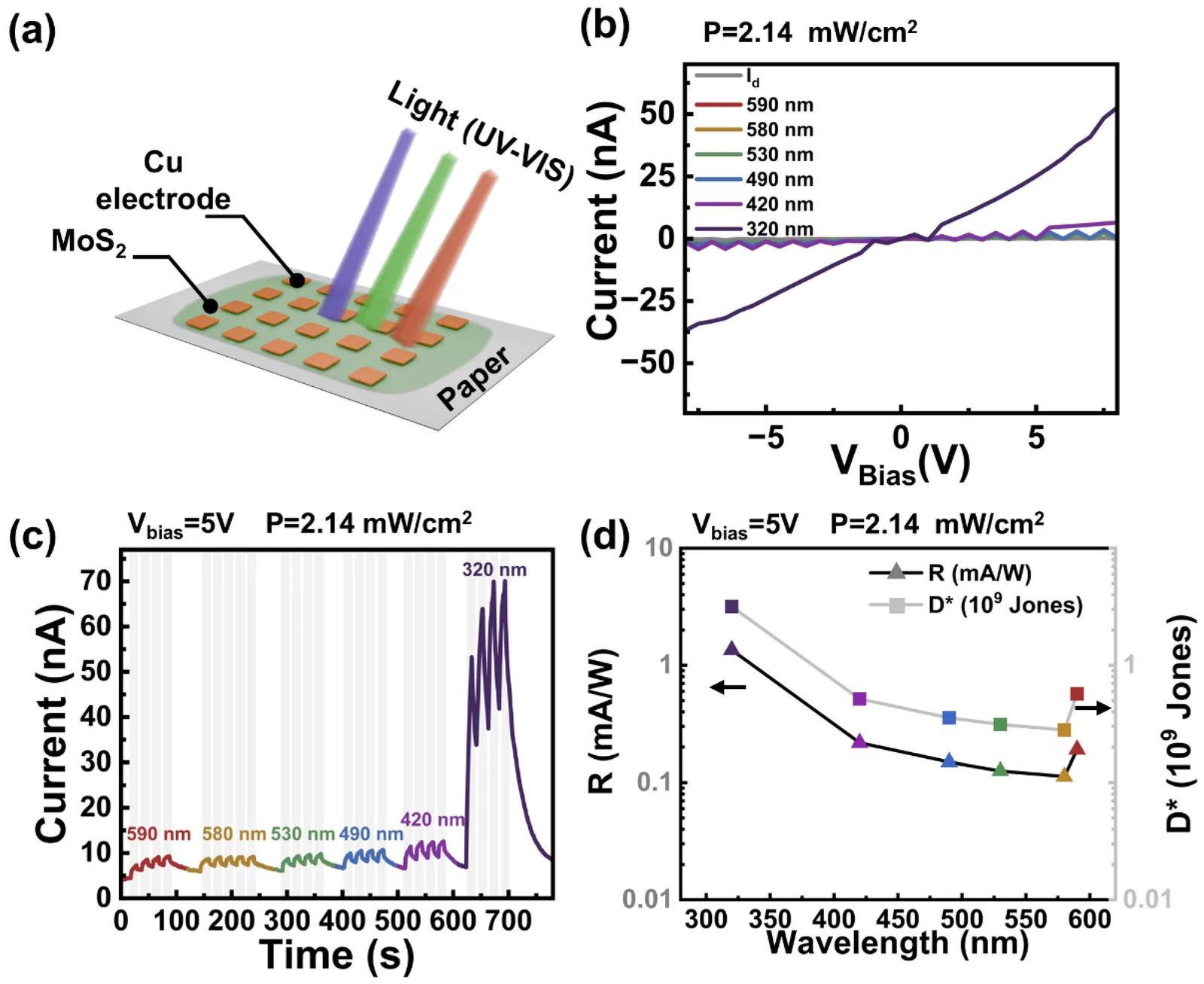
Electrical measurements of MoS_2_ paper-based photodetector under broadband illumination. (**a**) Schematic representation of the device on the paper substrate. (**b**) I–V measurements of the different wavelengths (320, 420, 490, 530, 580, 590) nm. (**c**) I–T measurements at the corresponding wavelengths. (**d**) Responsivity and specific detectivity of the MoS_2_ paper-based photodetector corresponding to the wavelength. The dot colors in (**d**) correspond to the wavelengths in (**c**). Panels (**c**,**d**) were measured at *P* = 2.14 mW/cm^2^ and a bias of 5 V.

The electrical measurements presented in Figure 6 further characterize the performance of the paper-based device. Figure 6a shows a schematic illustration of the device on the paper substrate. Figure 6b depicts the current–voltage (I-V) curve of the device at various wavelengths with an incident power density of 2.14 mW/cm^2^. From this curve, it is clear that the device performs best at 320 nm compared to other wavelengths. This behavior is consistent with the absorption spectrum in Figure 4a, where MoS_2_ shows strong absorption in the UV region, associated with the C-exciton and the van Hove singularity, so that more photocarriers are generated at 320 nm. In all cases, exciting an electron to the conduction band requires a photon energy exceeding the MoS_2_ bandgap (1.8 eV) [29].

The I-T curve in Figure 6c shows different illumination conditions at a voltage of 5 V and a power density of 2.14 mW/cm^2^. While a measurable signal across all wavelengths confirms broadband detection, responsivity and detectivity are key figures of merit that quantify a detector’s ability to sense light and are calculated using Equations (2) and (3). The relationship between wavelength and responsivity and detectivity is shown in Figure 6d. The device exhibits a decrease in responsivity and detectivity with increasing wavelength, because fewer photons are absorbed as the photon energy approaches the MoS_2_ bandgap, reducing photocarrier generation. A similar trend has been reported in previous research [21,33,36,44]. The maximum responsivity and detectivity of the device were 1.37 mA/W and 3.17 × 10^9^ Jones, respectively, at 320 nm. In addition, the NEP value of the device was calculated to be 3.45 × 10^−11^ W/Hz^−1/2^ using Equation (4), this value indicates better detection of the light under low illumination.

### 3.4. Response Time, Trap States and Benchmarking

To characterize how quickly the device responds to illumination, its photocurrent transients were examined in detail. The individual peak shapes at each wavelength are presented in Figure 6c, which shows approximately the same peak shape for all wavelengths. A slight, gradual rise in the photocurrent is observed over the I-T measurements, which we attribute to trap states that capture photogenerated electrons. Because these trapped carriers are released only slowly, recombination is delayed and the photocurrent does not fall back immediately when the illumination is switched off, producing the gradual rise seen in the curve [29]. In practical terms, this trap-limited behavior sets the response-speed ceiling of the present device; the origin of the traps is examined below.

**Figure 7:**
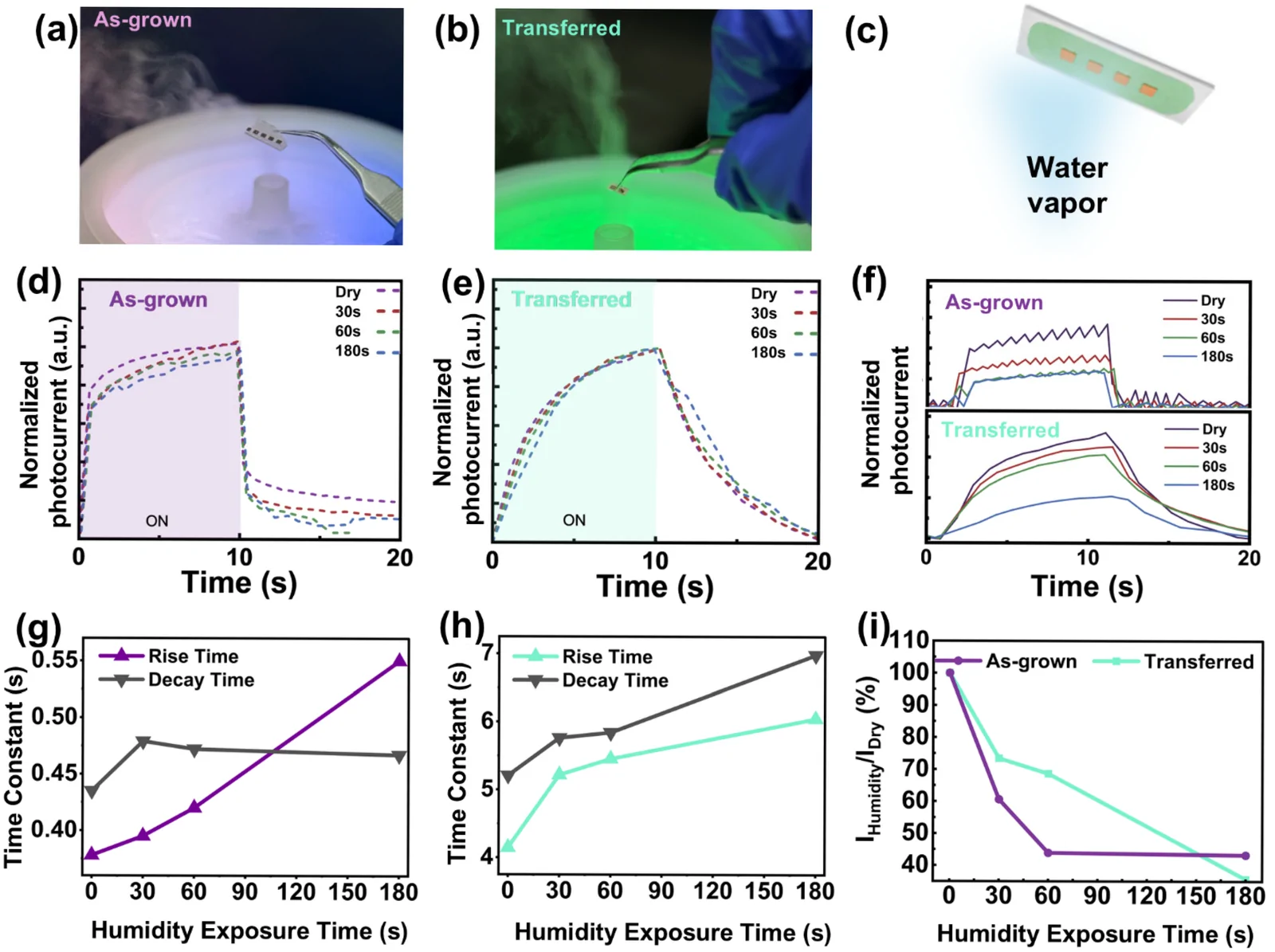
Humidity-dependent photoresponse of the as-grown and transferred MoS_2_ devices. (**a**,**b**) Photographs of the device held in the mist of an ultrasonic diffuser and (**c**) schematic of humidity exposure. (**d**,**e**) Normalized current-time transients of (**d**) the as-grown and (**e**) the transferred device under dry conditions and after humid exposures of 30 s, 60 s and 180 s. (**f**) Photocurrent transients of both devices under the same conditions. (**g**,**h**) Rise and decay times of (**g**) the as-grown and (**h**) the transferred device. (**i**) Photocurrent retained after each exposure. All measurements at λ = 320 nm, 5.5 mW/cm^2^ and 10 V.

To identify the origin of these traps, the as-grown and transferred devices were compared under dry conditions and after controlled humidity exposure (Figure 7). Both devices were exposed to water vapor for humid exposure times of 30 s, 60 s and 180 s (Figure 7a–c), and their normalized current-time transients were recorded after each step (Figure 7d,e). The rise time (τ_r_) is defined as the time for the signal to increase from 10% to 90% of its steady-state value, and the decay time (τ_d_) as the corresponding fall from 90% to 10%. Humidity reduces the photocurrent of both devices (Figure 7f,i) but it slows the response substantially only on paper (Figure 7g,h): under dry conditions the as-grown device on sapphire responds within approximately 0.4 s and changes by less than 0.2 s after humid exposure, whereas the rise and decay times of the transferred device on paper lengthen from 4.1 s and 5.2 s to 6.0 s and 7.0 s. Because both devices are fabricated from the same CVD film, and quantitative XPS gives essentially the same S:Mo ratio before and after transfer (2.01 and 2.03, Figure 3), intrinsic sulfur vacancies from growth cannot account for this order-of-magnitude slowing. The slow response is instead introduced by the transfer onto paper, through two extrinsic contributions: mechanical damage, evidenced by microcracks in a substrate-matched transfer control (Supplementary Figure S1) and by the low-energy photoluminescence tail extending to approximately 720 nm Figure 2d, which indicates localized band-tail states; and adsorbates, which the porous, hygroscopic paper takes up from the ambient and holds at the MoS_2_/paper interface, where they capture photogenerated carriers.

Because humidity slows the response only on paper, water adsorbed at the hygroscopic MoS_2_/paper interface introduces additional slow trap states. The same adsorbates also reduce the photocurrent by withdrawing electrons from the n-type MoS_2_ (Figure 7i). These interfacial traps also give rise to photogating gain, which, together with strain release (Section 3.2), accounts for the approximately 16-fold larger photocurrent of the transferred device and illustrates the familiar trade-off between gain and response speed.

### 3.5. Mechanical Flexibility and Bending Stability

**Figure 8:**
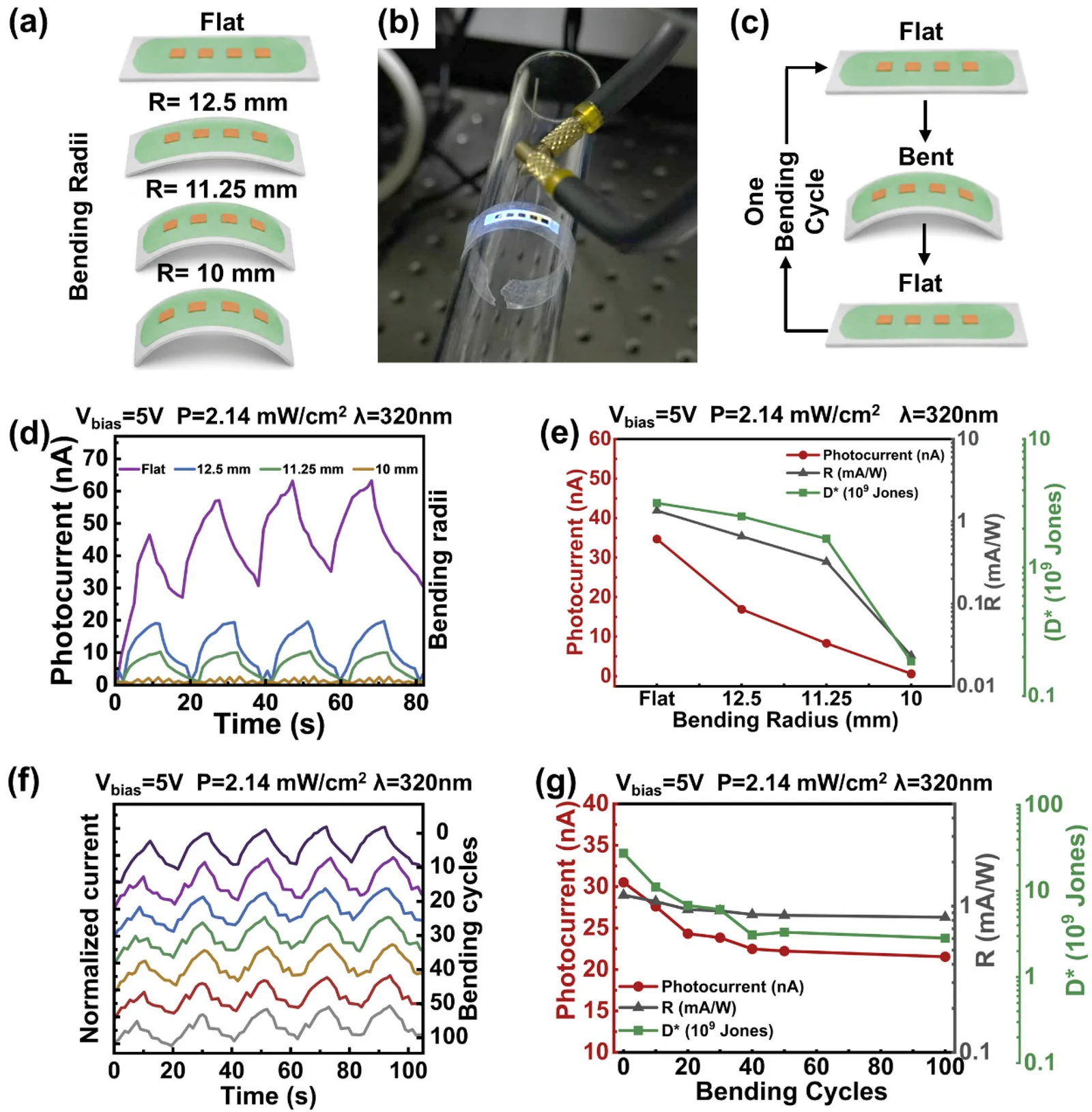
Mechanical flexibility of the paper-based photodetector. (**a**–**c**) Design of the bending experiments: (**a**) schematic of the flat state and of the bending radii of 12.5, 11.25 and 10 mm; (**b**) photograph of the device conformed to the curved former during measurement; (**c**) schematic of one bending cycle, defined as flat to bent and back to flat. (**d**,**e**) Bending-radius test: (**d**) current-time responses under chopped 320 nm illumination at each bending radius; (**e**) photocurrent, responsivity and specific detectivity as a function of bending radius. (**f**,**g**) Bending-cycle test: (**f**) normalized current-time responses after successive bending cycles up to 100 cycles, offset vertically for clarity (each color represents the number of cycles corresponding to the right axes); (**g**) photocurrent, responsivity and specific detectivity as a function of bending cycle number. All measurements at 5 V bias, 320 nm, and a power density of 2.14 mW/cm^2^.

The ability of a photodetector to maintain its functionality under bending is essential for its application in flexible electronics. To evaluate the mechanical flexibility of our proposed device, we therefore carried out two complementary tests, a bending-radius test and a bending-cycle test, the experimental design of which is summarized in Figure 8a–c. The device was bent from its flat initial state to radii of 12.5, 11.25 and 10 mm, as illustrated schematically in Figure 8a, with a photograph of the device conformed to the curved former during measurement shown in Figure 8b; one bending cycle, defined as flat to bent and back to flat, is illustrated in Figure 8c. In both tests the illuminated area itself was bent, so that the measurement reflects the true flexibility of the active device. Considering first the bending-radius test, the current-time responses measured under 320 nm illumination at an applied bias of 5 V for the different bending radii are shown in Figure 8d. The results indicate a decrease in the current upon decreasing the bending radius. While the device maintains a relatively stable response for the bending radii 12.5 and 11.25 mm, the performance at 10 mm becomes less stable. The figure of merits including photocurrent, responsivity and detectivity, are summarized in Figure 8e, where a gradual decrease in responsivity is observed as the device changes from the flat state to the bent state, consistent with the reduction in photocurrent under bending.

To further assess the mechanical robustness of the device, we subjected the device to repeated bending cycles at a radius of 12.5 mm, as shown in Figure 8f,g. Even under 100-bending cycles, our proposed paper photodetector still maintains a relatively stable current-time response as depicted in Figure 8f, which shows excellent bending robustness. The corresponding photocurrent, responsivity and detectivity are summarized in Figure 8g. These parameters exhibit a small variation over the investigated bending cycles, indicating the device stability under repeated mechanical deformation. After repeated bending, minor cracks were observed in the Cu film, but no delamination or detachment of the electrodes from the paper substrate occurred, indicating that the gradual decrease in photocurrent originates from electrode cracking rather than loss of adhesion. Taken together, these results show that our MoS_2_ paper-based photodetector continues to operate both under bending and after repeated deformation, which is significant for its role in wearable and flexible applications. Finally, the device performance is compared with previously reported MoS_2_ photodetectors in Table 1.

Table 1 compares the present device with representative MoS_2_ photodetectors reported to date. Because these devices differ markedly in film thickness, substrate, and contact metal, their raw figures of merit cannot be compared on a strict like-for-like basis. The highest responsivities in Table 1 are obtained either from multilayer or nanostructured MoS_2_, which has stronger light absorption than an atomically thin monolayer, or from monolayer devices fabricated on rigid sapphire with noble-metal (Au, Ag) contacts. In contrast, our device demonstrates better detection at low illumination, with high detectivity compared with the Cu-electrode device at an illumination power density of 75 mW/cm^2^. Moreover, the present work brings together three cost- and sustainability-oriented features in a single platform: a true CVD-grown monolayer, a flexible paper substrate, and a non-noble copper electrode. To the best of our knowledge, this combination has not been reported previously, and the responsivity is the expected trade-off for the reduced optical absorption of a single atomic layer together with a low-cost substrate and contact, rather than a limitation of the fabrication route itself. The device is therefore positioned not as a competitor to high-responsivity laboratory detectors, but as a step towards scalable and environmentally sustainable 2D optoelectronics.

## 4. Conclusions

In conclusion, this study demonstrates an integrated broadband UV–visible photodetector that combines a CVD-grown monolayer MoS_2_, a paper substrate, and a cost-effective copper electrode. Raman, photoluminescence, and X-ray photoelectron spectroscopy confirmed the high crystalline quality of the monolayer, while the paper substrate provided a flexible, low-cost, and sustainable platform, and the Cu electrode offered a practical alternative to conventional noble-metal contacts. The resulting device exhibited a broadband photoresponse across the UV and visible regions, with its best performance achieved under 320 nm illumination, reaching a maximum responsivity of 1.37 mA/W and a detectivity of 3.17 × 10^9^ Jones. Together, these results establish CVD-grown monolayer MoS_2_ on paper with a Cu electrode as a cost-effective, scalable route toward flexible, sustainable optoelectronics. The device retains a well-resolved photoresponse down to a bending radius of 11.25 mm and after 100 flat–bent–flat cycles, confirming that the paper platform tolerates the mechanical deformation relevant to practical use; realizing the full potential of this platform will require directly probing the proposed enhancement mechanisms.

## Figures and Tables

**Table 1:** Comparative results on MoS_2_ photodetectors.

Material	Substrate	Electrode	Spectral Range	Growth	R (mA/W)	D* (10^8^ Jones)	Other Parameter	Year/Ref
MoS_2_ Trilayer	Sapphire	Ag/Au	UV–Vis	CVD	8.6	2900	V = 1.5 V *P* = 0.6 µW/mm^2^ λ = 250 nm	2022 [21]
MoS_2_	Paper	PEDOT:PSS	UV–Vis	Vacuum filtering	0.13	-	V = 5 V *P* = 6.21 mW/cm^2^ λ = 405 nm	2019 [32]
MoS_2_	Paper	Cu tape	Vis	Mechanical rubbing	0.0016	-	V = 21 V *P* = 75 mW/cm^2^ λ = 660 nm	2020 [31]
MoS_2_ Monolayer	Sapphire	Ti/Au	UV–Vis	CVD	8.4	100	V = 0.5 V *P* = 0.051 mW/cm^2^ λ = 532 nm	2019 [20]
MoS_2_	PVDF filter membrane	Cu	UV–Vis	Vacuum filtering	963	290	V = 1 V *P* = 1.77 mW/cm^2^ λ = 405 nm	2020 [33]
MoS_2_	Mica	Cu	Vis	Ultrasonic-exfoliation	0.008	0.4	V = 1 V *P* = 1.77 mW/cm^2^ λ = 660 nm	2020 [45]
MoS_2_ Monolayer	Paper	Cu	UV–Vis	CVD	1.37	31.7	V = 5 V *P* = 2.14 mW/cm^2^ λ = 320 nm	This Work

## Data Availability

The original contributions presented in this study are included in the article/Supplementary Materials. Further inquiries can be directed to the corresponding authors.

## Supplementary Materials

The following supporting information can be downloaded at: https://www.mdpi.com/article/10.3390/mi17101156/s1, Figure S1. Substrate-matched transfer control. (a) Schematic of the PDMS-assisted transfer of the as-grown MoS_2_ from sapphire onto a fresh sapphire substrate. (b) Optical images of the as-grown and transferred MoS_2_; the arrow marks a crack in the transferred film. (c) Raman spectra of the as-grown and transferred MoS_2_, both on sapphire. Figure S2. Influence of adjacent structures. (a) Device before (Array) and after (Non-array) cutting to a single electrode pair; scale bar, 5 mm. (b) I-V characteristics in the dark and under 320 nm illumination. (c) I-T response under periodic 320 nm illumination at 5 V. *P* = 2.14 mW/cm^2^ in (b,c). Figure S3. Bare-paper control measurement. Current-time response under chopped 320 nm illumination at 2.14 mW/cm^2^ and 3 V bias of the identical Cu electrode structure on bare paper without MoS_2_, showing no response to illumination.

## Abbreviations

The following abbreviations are used in this manuscript:

2D	Two-dimensional
CVD	Chemical vapor deposition
TMDs	Transition metal dichalcogenides
PDMS	Polydimethylsiloxane
PL	Photoluminescence
XPS	X-ray photoelectron spectroscopy
UV–Vis	Ultraviolet–visible
FWHM	Full-width at half maximum
NEP	Noise equivalent power
PDCR	The corresponding photo-to-dark current ratio
PCC	persistent photoconductivity
