# Supplementary material for: Monolayer MoS2 on Paper with a Copper Electrode: An Integrated CVD-Grown Broadband UV–Visible Photodetector
Source: Micromachines (Basel). 2026 Sep 30;17(10):1156. doi: 10.3390/mi17101156 (PMC13707303; doi:10.3390/mi17101156)
Supplement: Supplementary file 1 [file micromachines-17-01156-s001.zip › micromachines-4565408-supplementary.pdf]

Supplementary Figures

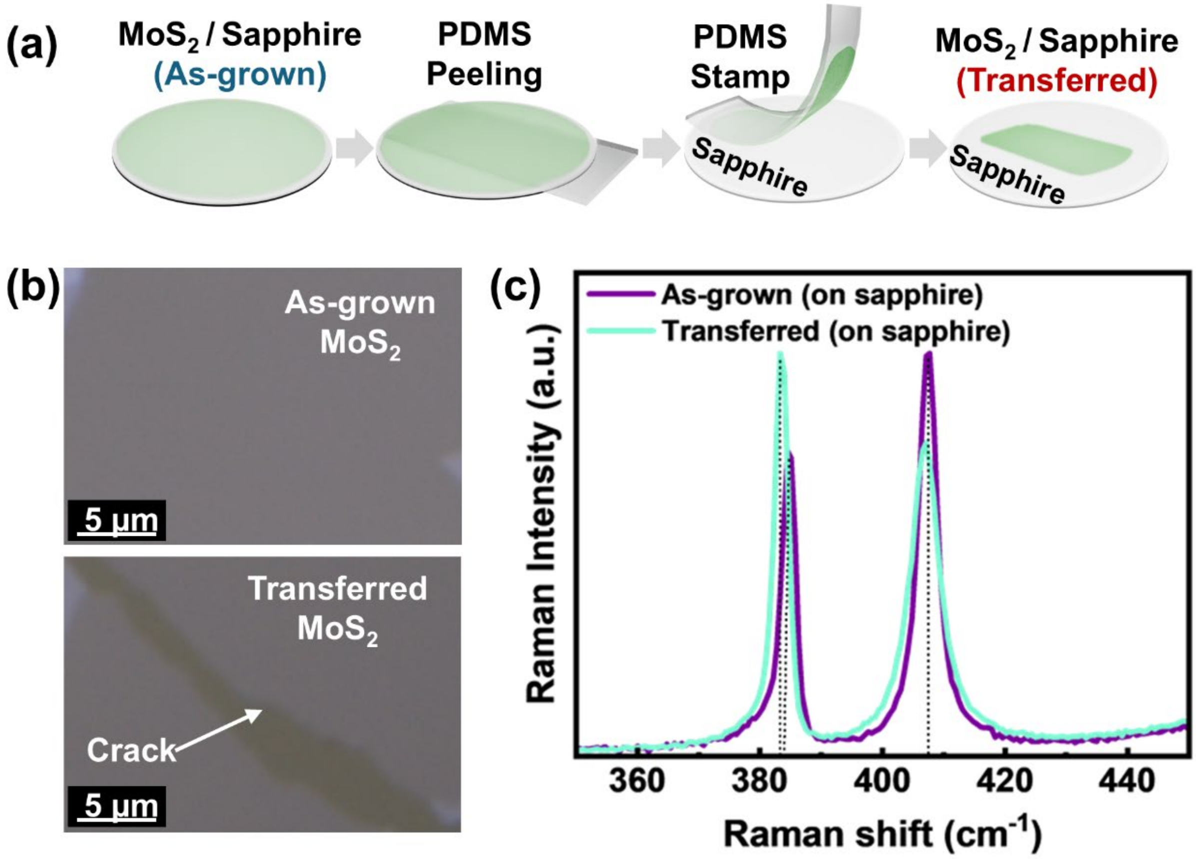

Figure S1. Substrate-matched transfer control. (a) Schematic of the PDMS-assisted transfer of the as-grown MoS_2 from sapphire onto a fresh sapphire substrate. (b) Optical images of the as-grown and transferred MoS_2; the arrow marks a crack in the transferred film. (c) Raman spectra of the as-grown and transferred MoS_2, both on sapphire.

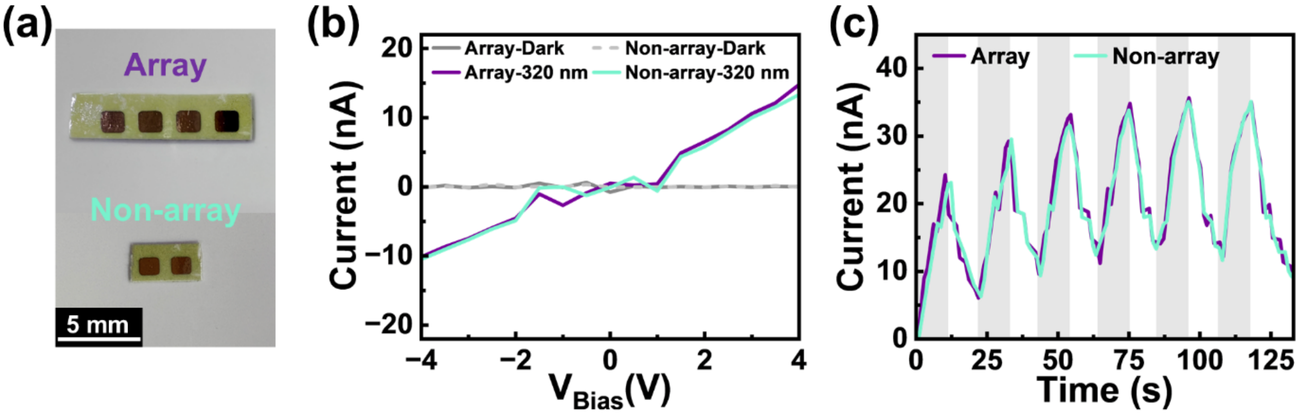

Figure S2. Influence of adjacent structures. (a) Device before (Array) and after (Non-array) cutting to a single electrode pair; scale bar, 5 mm. (b) I-V characteristics in the dark and under 320 nm illumination. (c) I-T response under periodic 320 nm illumination at 5 V. P = 2.14 mW/cm^2 in (b) and (c).

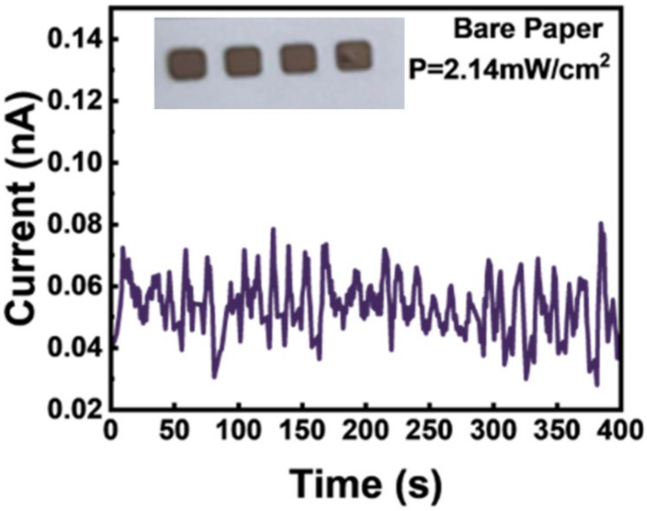

Figure S3. Bare-paper control measurement. Current-time response under chopped 320 nm illumination at 2.14 mW/cm² and 3 V bias of the identical Cu electrode structure on bare paper without MoS_2, showing no response to illumination.
